# Cooking skills related to potential benefits for dietary behaviors and weight status among older Japanese men and women: a cross-sectional study from the JAGES

**DOI:** 10.1186/s12966-020-00986-9

**Published:** 2020-06-26

**Authors:** Yukako Tani, Takeo Fujiwara, Katsunori Kondo

**Affiliations:** 1 Department of Global Health Promotion, Tokyo Medical and Dental University (TMDU), 1-5-45, Yushima, Bunkyo-ku, Tokyo, 113-8519 Japan; 2 Center for Preventive Medical Sciences, Chiba University, 1-8-1 Inohana, Chuo-ku, Chiba-shi, Chiba, 260-8672 Japan; 3 Department of Gerontological Evaluation, Center for Gerontology and Social Scienc, National Center for Geriatrics and Gerontology, 7-430 Morikoka-cho, Obu-shi, Aichi 474-8511 Japan

**Keywords:** Cooking skill, Home cooking, Eating out, Vegetable and fruit intake, Obesity, Underweight

## Abstract

**Background:**

Poor cooking skills have been linked to unhealthy diets. However, limited research has examined associations of cooking skills with older adults’ health outcomes. We examined whether cooking skills were associated with dietary behaviors and body weight among older people in Japan.

**Methods:**

We used cross-sectional data from the 2016 Japan Gerontological Evaluation Study, a self-report, population-based questionnaire study of men (*n* = 9143) and women (*n* = 10,595) aged ≥65 years. The cooking skills scale, which comprises seven items with good reliability, was modified for use in Japan. We calculated adjusted relative risk ratios of unhealthy dietary behaviors (low frequency of home cooking, vegetable/fruit intake; high frequency of eating outside the home) using logistic or Poisson regression, and relative risk ratios of obesity and underweight using multinomial logistic regression.

**Results:**

Women had higher levels of cooking skills, compared with men. Women with a moderate to low level of cooking skills were 3.35 (95% confidence interval [CI]: 2.87–3.92) times more likely to have a lower frequency of home cooking and 1.61 (95% CI: 1.36–1.91) times more likely to have a lower frequency of vegetable/fruit intake, compared with women with a high level of cooking skills. Men with a low level of cooking skills were 2.56 (95% CI: 2.36–2.77) times more likely to have a lower frequency of home cooking and 1.43 (95% CI: 1.06–1.92) times more likely to be underweight, compared with men with a high level of cooking skills. Among men in charge of meals, those with a low level of cooking skills were 7.85 (95% CI: 6.04–10.21) times more likely to have a lower frequency of home cooking, 2.28 (95% CI: 1.36–3.82) times more likely to have a higher frequency of eating outside the home, and 2.79 (95% CI: 1.45–5.36) times more likely to be underweight, compared with men with a high level of cooking skills. Cooking skills were unassociated with obesity.

**Conclusions:**

A low level of cooking skills was associated with unhealthy dietary behaviors and underweight, especially among men in charge of meals. Research on improving cooking skills among older adults is needed.

## Background

There are increasing calls to return to home cooking to prevent poor diets and chronic diet-related diseases [1]. A systematic review has reported the dietary benefits of eating home-cooked meals, including greater consumption of fruits and vegetables, enhanced nutrient intake, and higher diet quality [2]. A recent cross-sectional study showed that eating home-cooked dinners was associated with greater dietary guideline compliance, without significantly increasing food expenditures [3]. Although studies related to the effects of home cooking on health outcomes are limited, a recent large population-based study in the United Kingdom showed that more frequent consumption of home-cooked meals was associated with a greater likelihood of having normal weight and body fat status [4]. Furthermore, a cohort study targeting older people in Taiwan demonstrated that older adults who cooked more than five times per week had approximately 40% lower risk of death, compared with those who did not cook [5]. The study also showed a dose–response relationship, meaning that the risk of death decreased as the frequency of home cooking increased. Despite the benefits of home cooking, the consumption of home-cooked meals has declined and the consumption of out-of-home foods, such as fast food and convenience food, has increased in recent decades in developed countries [6, 7].

Cooking skills are one important modifiable factor that can encourage people to cook [2]. In addition to increasing the frequency of home cooking, strengthening people’s cooking skills can improve their diet quality. For example, cross-sectional studies have shown an association between high levels of cooking skills and lower consumption of ready meals, convenience food, and ultra-processed food among adults [8–10]. Intervention studies have also shown improving cooking skills to increase cooking confidence and consumption of vegetables and fruits [11, 12]. Most existing studies have focused on dietary benefits among adults, and limited work has examined the associations between cooking skills and health outcomes among older adults.

Population aging is increasing dramatically, and the percentage of the world’s population aged over 60 years is projected to nearly double from 12% in 2015 to 22% in 2050 [13]. Overall, older adults do not meet the recommendations for a healthy diet [14]. Physiological, social, economic, and psychological factors affect older people’s food choices. Physiological factors, such as age-related decline in taste and smell, can lead to decreased appetite and poor dietary habits [15]. Social factors, including lower social engagement and living alone, are also associated with poor diet quality [15, 16]. Economic factors such as low income and retirement can negate older people’s ability to meet their nutritional needs [17, 18]. Finally, psychological factors including wellbeing and depression are also associated with eating behaviors [16]. However, although cooking skills are a fundamental factor in preparing meals, the effects of cooking skills on dietary behaviors do not seem to have been evaluated among older adults. A systematic review demonstrated associations between culinary interventions and improved dietary factors, including attitudes, self-efficacy, and healthy dietary intake among adults [19]. Focusing on cooking skills as a modifiable factor among older adults is an innovative approach.

The rationales described above indicate that having sufficient cooking skills may be important for healthy aging. More older adults live alone, compared with other age groups: In 43 developing countries, only 1.6% of people were found to live alone overall, compared with 8.8% of older adults [20]. In Japan, most older people live alone or with their spouses [21]. Thus, older adults are faced with the task of preparing own meals. Because of changes in living arrangements or spouses becoming unable to cook, the person responsible for cooking at home may change in older age. For example, a widowed man may be in charge of cooking for the first time. Because it is mainly women who are in charge of preparing meals, men have been found to be less confident in their cooking and to have lower levels of cooking skills [9, 22]. Therefore, men, especially widowed men or men whose spouses are unable to cook, may be at risk of diet-related problems because of their poor cooking skills. To our knowledge, no study has examined the associations between cooking skills and health outcomes among older people who are in charge of meals.

One reason for the limited evidence relating to cooking skills may be the difficulty of assessing cooking skills. Cooking skills have been defined as a set of mechanical or physical skills used in meal preparation, such as chopping, mixing, and heating basic ingredients, as well as conceptual skills related to understanding how food will react when cooked [23]. In addition to the various aspects of cooking skills, the cooking skills required vary depending on culture: For example, cooking methods (e.g., grilling, steaming, stewing, and stir-frying) differ by culture. Several methods have been used to measure cooking skills, but there are few validated and reliable measures of cooking skills [23]. Hartmann et al. conducted a test-retest analysis and designed a reliable cooking skills scale comprising seven items related to the ability to prepare different foods that is applicable to most European cultures [9]. Because this scale rates the ability to prepare general food groups (e.g., bread), it is more versatile than scales that rate the ability to prepare specific meals (e.g., spaghetti Bolognese). Therefore, for the present study, we modified this scale for application in a Japanese population. The first aim of our study was to assess the reliability of this scale in a large-scale Japanese population-based study. The second aim was to examine the associations of cooking skills with the frequency of home cooking, the frequency of eating outside the home, the frequency of vegetable/fruit consumption, and body weight status.

## Methods

### Study design and participants

The Japan Gerontological Evaluation Study (JAGES), a large nation-wide research project on aging, was established in 2010 to evaluate the social determinants of healthy aging among older people in Japan [24, 25]. We used data from the 2016 JAGES, which covered 39 municipalities across Japan and was administered to community-dwelling older adults who were physically and cognitively independent (i.e., without functional disabilities, defined as not being certified as eligible to receive long-term public care insurance system services [26]). From October 2016 to January 2017, self-report questionnaires were mailed to 279,661 older adults aged ≥65 years. The survey was conducted using random sampling in 22 large municipalities and was administered to all eligible residents in 17 small municipalities. A total of 196,438 participants returned the questionnaire (response rate: 70.2%). In some municipalities, recipients receiving long-term public care insurance benefits were included in the survey by request of the local government, so the target sample was 180,021 older adults, after excluding those who received these benefits. One-eighth of the sample (*N* = 22,219) were randomly selected to receive a survey module on cooking skills. The present analysis was carried out using data for 19,738 participants (9143 men and 10,595 women), after the following exclusions: participants with missing information on gender (*N* = 2); participants who did not complete the questions related to height and weight (*N* = 660) or dietary behaviors (frequency of home cooking, eating outside the home, and vegetable/fruit intake) (*N* = 1475); participants with missing data on the cooking skills scale (*N* = 145); and participants who were included in this study accidentally who reported limitations in activities of daily living (*N* = 199) to ensure that the sample was actually physically and cognitively independent. Limitations in activities of daily living were assessed with the Independence in Activities of Daily Living index [27] using the following questionnaire item: “Do you need any nursing care or assistance from someone in your daily life?” We excluded participants who answered “I need and receive nursing care or assistance.” Participants were informed that participation in the study was voluntary and that completing and returning the questionnaire indicated their consent to participate in the study. The JAGES protocol was approved by the Ethics Committee in Research of Human Subjects at the National Center for Geriatrics and Gerontology (No. 992) and Chiba University Faculty of Medicine (No. 2493).

### Body weight status and dietary behaviors

Participants reported their height in centimeters and weight in kilograms. Body mass index (BMI) was calculated as weight divided by the square of height (kg/m^2^). We defined underweight as having a BMI < 18.5 kg/m^2^ and obesity as having a BMI ≥ 27.5 kg/m^2^, following the suggested cutoff points for Asians [28]. The evaluated dietary behaviors were the frequency of home cooking, eating outside the home, and vegetable/fruit intake. The frequency of home cooking was assessed using the question “How often do you cook by yourself? Do not include ready-to-eat food” (responses: *more than five times a week*, *three to five times a week*, *one to two times a week*, *less than once a week*, and *never*). Respondents who cooked less than two times a week were categorized as having a low cooking frequency for women because more than three times a week has been shown to predict survival among older women [5]. For men, respondents who never cooked were categorized as having a low cooking frequency because more than half of the men indicated that they never cooked (Table 1). The frequency of eating outside the home was assessed using the question “How often do you eat outside the home?” The responses for this item were the same as those for the frequency of home cooking. Respondents who ate outside the home more than three times a week were categorized as having a high frequency of eating outside the home because eating outside the home more than three times a week has been shown to be related to higher BMI and lower serum concentrations of nutrients [29]. The frequency of vegetable and fruit intake was assessed using the question “How often did you eat vegetables and fruits over the past month?” (responses: *not at all*, *less than once a week*, *once a week*, *two to three times a week*, *four to six times a week*, *once a day*, and *at least twice a day*) [30, 31]. Respondents who ate vegetables and fruits less than once a day were categorized as having a low frequency of vegetable and fruit intake. This cutoff point was defined by prevalence to be under 25% of subjects included (Table 1) because being in the lowest quartile for vegetable and fruit intake has been shown to be associated with poor health outcomes [32–34].

**Table 1:** Characteristics of older Japanese men and women by level of cooking skills (*n* = 19,378)

	Males (*n* = 9143)						Females (*n* = 10,595)				
	Total		Cooking skill				Total		Cooking skill		
	n	%	High	Middle	Low	*p*-value	n	%	High	Middle/Low	*p*-value
			%	%	%				%	%	
Cooking skill											
High	4751	52.0	100	0	0		9968	94.1	100.0	0	
Middle	3269	35.8	0	100	0		498	4.7	0	79.4	
Low	1123	12.3	0	0	100		129	1.2	0	20.6	
Age (years)											
65–69	2855	31.2	32.7	29.8	29.2	< 0.001	3269	30.9	31.7	18.0	< 0.001
70–74	2510	27.5	26.9	29.1	25.1		2893	27.3	27.8	19.9	
75–79	2059	22.5	22.9	22.3	21.4		2393	22.6	22.8	19.8	
≥ 80	1719	18.8	17.5	18.8	24.3		2040	19.3	17.8	42.3	
Education (years)											
Low (≤9)	2626	28.7	28.2	28.1	32.7	0.002	3614	34.1	33.3	46.4	< 0.001
Middle (10–12)	3461	37.9	36.8	39.4	37.9		4638	43.8	44.2	37.0	
High (≥13)	2981	32.6	34.1	31.8	28.8		2198	20.7	21.1	14.7	
Other/Missing	75	0.8	0.9	0.8	0.6		145	1.4	1.3	1.9	
Annual income (million yen)											
Low (< 2.00)	3485	38.1	37.5	38.6	39.4	0.215	4110	38.8	38.5	43.1	< 0.001
Middle (2.00–3.99)	3279	35.9	35.9	36.6	33.3		3049	28.8	29.3	21.1	
High (≥4.00)	924	10.1	10.6	9.3	10.6		885	8.4	8.5	5.9	
Missing	1455	15.9	16	15.4	16.7		2551	24.1	23.7	30.0	
Marital status											
Married	7788	85.2	81.6	88.0	92.0	< 0.001	6497	61.3	62.4	44.7	< 0.001
Widowed	631	6.9	8.5	5.4	4.3		3097	29.2	28.3	43.9	
Divorced	321	3.5	4.7	2.6	1.2		513	4.8	4.8	5.1	
Single	257	2.8	3.4	2.6	1.0		307	2.9	2.8	4.1	
Other/Missing	146	1.6	1.8	1.3	1.5		181	1.7	1.7	2.2	
Under medical treatment											
Cancer (Yes)	450	4.9	5.1	4.5	5.7	0.53	355	3.4	3.3	4.3	0.35
Heart disease (Yes)	1219	13.3	13	13.6	14.2	0.84	713	6.7	6.5	9.7	0.008
Stroke (Yes)	385	4.2	3.5	4.5	6.5	< 0.001	184	1.7	1.6	4.1	< 0.001
Diabetes mellitus (Yes)	1484	16.2	16.6	15.3	17.4	0.47	1010	9.5	9.5	10.4	0.69
Hypertension (Yes)	3913	42.8	42.1	43.5	43.8	0.66	4391	41.4	41.0	48.2	0.002
Hyperlipidemia (Yes)	933	10.2	10.3	10.4	9.4	0.92	1658	15.6	15.8	13.2	0.19
Main way of preparing meals											
Cook by yourself	1283	14.0	22.2	6.1	2.2	< 0.001	8714	82.2	84.0	54.9	< 0.001
Family member cook	6785	74.2	65.1	82.5	88.5		977	9.2	7.9	30.3	
Buy cooked meals	307	3.4	2.8	4.3	2.8		132	1.2	1.1	4.1	
Use home-delivery services	81	0.9	0.9	0.7	1.4		46	0.4	0.4	1.0	
Other/Missing	687	7.5	8.9	6.3	5.1		726	6.9	6.7	9.7	
Frequency of home cooking (n/week)											
≥ 5 /week	1310	14.3	23.3	5.8	1.2	< 0.001	8762	82.7	84.6	51.8	< 0.001
3–4 /week	757	8.3	12.5	4.6	1.1		902	8.5	8.3	11.8	
1–2 /week	1002	11	15.3	7.7	2.0		366	3.5	3.2	8.1	
< 1 /week	1157	12.7	15.2	12.4	2.8		199	1.9	1.5	7.2	
Never	4917	53.8	33.7	69.6	92.9		366	3.5	2.3	21.1	
Frequency of eating outside the home (n/week)											
≥ 5 /week	242	2.6	2.6	2.3	3.7	< 0.001	103	1.0	0.9	1.4	0.001
3–4 /week	462	5.1	5.0	5.4	4.2		268	2.5	2.6	2.1	
1–2 /week	1450	15.9	16.9	15.6	12.3		1268	12.0	12.1	10.4	
< 1 /week	3773	41.3	41.3	41.9	39.2		4297	40.6	41.0	34.1	
Never	3216	35.2	34.2	34.7	40.7		4659	44.0	43.5	52.0	
Frequency of vegetable/fruit intake (n/day)											
≥ 1 /day	6625	72.5	73.3	70.6	74.4	0.008	9052	85.4	86.0	75.9	< 0.001
< 1 /day (low frequency)	2518	27.5	26.7	29.4	25.6		1543	14.6	14.0	24.1	
Body weight status (BMI, kg/m^2^)											
Underweight (< 18.5)	428	4.7	4.1	5.1	5.8	0.04	988	9.3	9.2	11.8	0.08
Normal (18.5–27.4)	8044	88.0	88.1	88.0	87.4		8833	83.4	83.5	81.5	
Obesity (≥ 27.5)	671	7.3	7.8	6.9	6.9		774	7.3	7.3	6.7	

*BMI* body mass index

### Cooking skills

As mentioned above, based on the cooking skills scale for European cultural regions [9], we adapted Hartmann’s a cooking skills scale for use in Japanese populations. In Japan, a typical meal—known as *ichi-ju san-sai*—consists of a staple food (such as rice), a soup (usually miso), and three dishes (one main dish and two side dishes) [35]. The basic Japanese cooking methods—*Gohou* (five methods)—are raw food, boiling, grilling, steaming, and frying [36]. We adopted stewing instead of steaming to reflect contemporary cooking practices [37]. Therefore, we included these elements and designed the following seven items for the Japanese version of the cooking skills scale: “How do you assess your overall cooking skills?”; “Can you peel fruits and vegetables?”; “Can you boil eggs and vegetables?”; “Can you grill fish?”; “Can you make stir-fried meat and vegetables?”; “Can you make miso soup?”; and “Can you make stewed dishes?” Participants were asked to evaluate their own cooking skills on a six-point scale (ranging from 1 for *unable* to 6 for *very well*). Cronbach’s α for these seven items was 0.96. Cronbach’s α was calculated using an unstandardized approach for respondents answering five or more of the seven items. The mean of the seven items was calculated for each respondent to reflect their overall cooking skills; the midpoint was 3.5, and a high score meant that the respondent had high confidence in their cooking skills (Table 2). The mean cooking skills score was divided into three categories—high (> 4.0), middle (2.1–4.0), and low (≤ 2.0)—to examine the associations of cooking skills with body weight status and dietary behaviors. For women, because the distribution of the cooking skills score was skewed to the left (leaning towards higher scores), the middle and low groups were merged into one category. Therefore, women were classified into two cooking skills categories: high (> 4.0) and middle/low (≤ 4.0).

### Person in charge of meal selection

Participants were asked “In what way are your daily meals mainly prepared?” The responses to this item were as follows: *cook by myself*, *a family member cooks*, *buy packaged lunches or cooked meals*, *use catering or home-delivery services*, and *other*. Participants except for those who reported that a family member did the cooking were defined as being in charge of preparing or selecting meals.

### Covariates

Covariates were assessed using the self-report questionnaire. Age was divided into four categories (65–69, 70–74, 75–79, and ≥ 80 years). The duration of education was divided into three categories (≤ 9 years, 10–12 years, and ≥ 13 years). Annual household income was adjusted for household size, dividing the household income by the square root of the number of people in the household. This variable was then divided into three categories (< 2.00, 2.00–3.99, and ≥ 4.00 million yen). Marital status was divided into five categories (married, widowed, divorced, single, and other). To assess comorbidity, the participants were asked whether they were currently under medical treatment for any of the following conditions (multiple responses were allowed): cancer, heart disease, stroke, hypertension, diabetes mellitus, and hyperlipidemia. Covariates with missing data were categorized as “missing.”

### Statistical analysis

The analyses were stratified by gender because a previous study reported different associations between cooking skills and dietary behaviors by gender and distinct patterns of potential confounders for men and women [9]. First, participants were stratified by cooking skill level, and differences between groups were tested using Pearson’s chi-squared tests. Second, multiple comparisons for the cooking skills scale were analyzed using the mixed linear model procedure to examine which cooking skills participants rated as difficult. The model adjusted for age, education, annual normalized household income, marital status, and medical treatment (cancer, heart disease, stroke, diabetes mellitus, hypertension, and hyperlipidemia), and peeling was used as the reference category. Participant identification code was included as a random effect. Third, we calculated adjusted odds ratios with 95% confidence intervals (CIs) of high frequency of eating outside the home using logistic regression. For low frequency of home cooking and vegetable/fruit intake, we calculated adjusted prevalence ratios (APRs) with 95% CIs using Poisson regression because participants with low frequencies of home cooking and vegetable/fruit intake were not uncommon, so the odds ratios derived from logistic regression would have been unable to approximate the prevalence ratio [38, 39]. For the association with weight status, we calculated adjusted relative risk ratios (ARRRs) with 95% CIs of underweight and obesity using multinomial logistic regression, with the body weight category of BMI of 18.5–27.4 kg/m^2^ as the reference category. The models were adjusted for the following potential confounding factors: age, education, annual normalized household income, and medical treatment for cancer, heart disease, stroke, hypertension, diabetes mellitus, and hyperlipidemia. Participants with missing data on the covariates were included in the analysis. All analyses were conducted using Stata, Version 14 (Stata Statistical Software: Release 14. College Station, TX: StataCorp LP).

## Results

The participants’ characteristics are summarized in Table 1. A total of 46% of the participants were men, about 20% were aged over 80 years, 30% had under 9 years of education, and 40% had annual incomes below two million yen. Of the male respondents, about 10% were widowed or divorced. When cognitive function was assessed with three items from the Kihon Checklist–Cognitive Function scale, for which predictive validity for dementia incidence has previously been confirmed [40], only 0.9% of participants had three cognitive complaints. The majority of women (94.1%) were classified as having a high level of cooking skills (Table 1). For men, the level of cooking skills was classified as high for 52.0%, middle for 35.8%, and low for 12.3%. For women, 8.9% cooked less than two times a week, 3.5% ate out more than three times a week, 14.6% ate vegetables/fruits less than once a day, 9.3% were underweight, and 7.3% were obese. For men, 53.8% never cooked, 7.7% ate out more than three times a week, 27.5% ate vegetables/fruits less than once a day, 4.7% were underweight, and 7.3% were obese. Women with middle/low-level cooking skills tended to be older, have a low level of education, have low income, not be married, and list a family member as the main meal preparer (Table 1). For men, in addition to being older, having a low level of education, and having a family member as the main meal preparer, men who were married tended to have a low level of cooking skills (Table 1).

The mean cooking skills score was higher for women (5.6 points) than for men (4.1 points) (t (19736) = − 99.6, *p* < 0.001) (Table 2). For psychometric testing, one factor with an eigenvalue over 1 was found, and this accounted for 80.5% of the variance. All factor loadings were 0.8 or higher. Men rated stewing and stir-frying as more difficult than peeling. Although women had statistically significant differences between the assessed cooking skills, in terms of substantive significance, they rated all the methods assessed on the cooking skills scale as being of similar difficulty (Table 2).

**Table 2:** Cooking skills among older Japanese men (*n* = 9143) and women (*n* = 10,595)

Items	Males				Females			
	mean	SD	Coef	*p*-value	mean	SD	Coef	*p*-value
Overall cooking skills	3.15	1.53	−1.60	< 0.001	4.99	1.12	−0.77	< 0.001
Able to peel fruits and vegetables	4.75	1.44	reference		5.76	0.74	reference	
Able to boil eggs and vegetables	4.58	1.56	−0.17	< 0.001	5.78	0.73	0.02	0.007
Able to grill fish	4.24	1.72	−0.50	< 0.001	5.70	0.86	−0.06	< 0.001
Able to make stir-fried meat and vegetables	4.04	1.76	−0.70	< 0.001	5.72	0.82	−0.04	< 0.001
Able to make miso soup	4.16	1.77	−0.59	< 0.001	5.74	0.80	−0.02	0.001
Able to make stewed dishes	3.43	1.81	−1.32	< 0.001	5.72	0.82	−0.05	< 0.001
Cooking skill scale	4.05	1.42			5.63	0.74		

Multiple comparisons between cooking skills scale were analyzed using the mixed linear models procedure adjusted for age, education, annual normalized household income, marital status and medical treatment (cancer, heart disease, stroke, diabetes mellitus, hypertension and hyperlipidemia). Participant identification code was included as a random effect

There were gender differences in the associations of cooking skills with unhealthy dietary behaviors and body weight status (Table 3). Women with a middle/low level of cooking skills were 3.35 times (95% CI: 2.87–3.92) more likely to have a lower frequency of home cooking and 1.61 (95% CI: 1.36–1.91) times more likely to have a lower frequency of vegetable/fruit intake, compared with women with a high level of cooking skills. As for weight status, women with a middle/low level of cooking skills were 1.29 (95% CI: 0.99–1.67) times more likely to be underweight, compared with women with a high level of cooking skills. For men, compared with those with a high level of cooking skills, men with a middle or low level of skill were 1.98 (95% CI: 1.86–2.11) times more likely and 2.56 (95% CI: 2.36–2.77) times more likely, respectively, to have a lower frequency of home cooking. Regarding eating outside the home, compared with men with a high level of cooking skills, men with a low level of cooking skills were 1.30 (95% CI: 1.01–1.67) times more likely to have a higher frequency of eating outside the home. There was a significant association with a low frequency of vegetable/fruit intake only among men with a middle level of cooking skills (APR: 1.15, 95% CI: 1.06–1.26). As for weight status, compared with men with a high level of cooking skills, men with middle or low skill levels were 1.29 (95% CI: 1.04–1.60) times more likely and 1.43 (95% CI: 1.06–1.92) times more likely, respectively, to be underweight. There was no significant association between cooking skills and obesity for either men (*p* = 0.33) or women (*p* = 0.40). Using the cutoff point of BMI ≥ 23.0 kg/m^2^ as overweight, we found that a low level of cooking skills was not associated with an increased risk of overweight (Supplementary Table 1).

**Table 3:** Adjusted prevalence/odds ratios/relative risk ratios of unhealthy eating behaviors, underweight, and obesity by level of cooking skills among older Japanese men (*n* = 9203) and women (*n* = 10,847)

		Low frequency of home cooking^a^	High frequency of eating outside the home^b^	Low frequency of vegetable and fruit intake^c^	Underweight^d^	Obesity^e^
		APR (95%CI)	AOR (95%CI)	APR (95%CI)	ARRR (95%CI)	ARRR (95%CI)
Men						
Cooking skill	High	ref	ref	ref		ref
	Middle	**1.98 (1.86–2.11)**	1.15 (0.97–1.36)	**1.15 (1.06–1.26)**	**1.29 (1.04–1.60)**	0.89 (0.75–1.06)
	Low	**2.56 (2.36–2.77)**	**1.30 (1.01–1.67)**	1.04 (0.91–1.18)	**1.43 (1.06–1.92)**	0.88 (0.68–1.14)
Women						
Cooking skill	High	ref	ref	ref		ref
	Middle-Low	**3.35 (2.87–3.92)**	0.97 (0.62–1.52)	**1.61 (1.36–1.91)**	1.29 (0.99–1.67)	0.87 (0.62–1.21)

*AOR* adjusted odds ratio, *APR* adjusted prevalence ratio, *ARRR* adjusted relative risk ratio, *BMI* body mass index, *CI* confidence interval

^a^Low frequency of home cooking: frequency of home cooking ≤2 times/week for women and 0 times/week for men

^b^High frequency of eating outside the home: frequency of eating outside the home ≥3 times/week

^c^Low frequency of vegetable and fruit intake: frequency of vegetable and fruit intake < 1/day

^d^Underweight: BMI < 18.5 kg/m^2^

^e^Obesity: BMI ≥ 27.5 kg/m^2^

The models were adjusted for age, education, annual normalized household income, marital status, and medical treatment (cancer, heart disease, stroke, diabetes mellitus, hypertension, and hyperlipidemia)

Next, we focused on men in charge of meals. Over 90% of women (*n* = 9618) but only 26% of men (*n* = 2358) were in charge of daily meals. In contrast to men not in charge of preparing meals, most men in charge of meals rated their cooking skills as high (Supplementary Table 2). Men in charge of meals tended to have low levels of education and low income and to be unmarried (e.g., widowed or divorced) (Supplementary Table 2). When the associations with unhealthy dietary behaviors and weight status were examined for men in charge of meals, the effect size increased (Table 4). Compared with men with a high level of cooking skills, men with middle- or low-level cooking skills were 4.22 (95% CI: 3.42–5.21) times more likely and 7.85 (95% CI: 6.04–10.21) times more likely, respectively, to have a lower frequency of home cooking (Table 4). Regarding eating outside the home, compared with men with a high level of cooking skills, men with a low level of cooking skills were 2.28 (95% CI: 1.36–3.82) times more likely to have a higher frequency of eating outside the home. In relation to low frequency of vegetable/fruit intake, the APR for men with a middle level of cooking skills was 1.32 (95% CI: 1.15–1.53). Furthermore, compared with men with a high level of cooking skills, men with middle or low skill levels were 1.59 (95% CI: 1.04–2.45) times more likely and 2.79 (95% CI: 1.45–5.36) times more likely, respectively, to be underweight.

**Table 4:** Adjusted prevalence ratios/relative risk ratios of low frequency of vegetable/fruit intake and home cooking, underweight, and obesity by cooking skills level among older Japanese men in charge of meals (*n* = 2370)

		Low frequency of home cooking^a^	High frequency of eating out^b^	Low frequency of vegetable and fruit intake^c^	Underweight^d^	Obesity^e^
		APR (95%CI)	AOR (95%CI)	APR (95%CI)	ARRR (95%CI)	ARRR (95%CI)
Men in charge of cooking						
Cooking skill	High	ref		ref	ref	ref
	Middle	**4.22 (3.42–5.21)**	**1.63 (1.22–2.17)**	**1.32 (1.15–1.53)**	**1.59 (1.04–2.45)**	0.91 (0.63–1.32)
	Low	**7.85 (6.04–10.21)**	**2.28 (1.36–3.82)**	1.12 (0.84–1.50)	**2.79 (1.45–5.36)**	0.59 (0.25–1.39)

*AOR* adjusted odds ratio, *APR* adjusted prevalence ratio, *ARRR* adjusted relative risk ratio, *BMI* body mass index, *CI* confidence interval

^a^Low frequency of home cooking: frequency of home cooking 0 times/week

^b^High frequency of eating outside the home: frequency of eating outside the home ≥3 times/week

^c^Low frequency of vegetable and fruit intake: frequency of vegetable and fruit intake < 1/day

^d^Underweight: BMI < 18.5 kg/m^2^

^e^Obesity: BMI ≥ 27.5 kg/m^2^

The models were adjusted for age, education, annual normalized household income, marital status, and medical treatment (cancer, heart disease, stroke, diabetes mellitus, hypertension, and hyperlipidemia)

## Discussion

To the best of our knowledge, this is the first study to examine the associations of cooking skills with unhealthy dietary behaviors and weight status by gender and meal preparer status among older adults. Using an adapted version of an existing cooking scale for use in Japanese populations, we confirmed that women had higher levels of cooking skills than did men and that the associations of cooking skills with dietary behaviors and weight status differed by gender. For both men and women, a low or middle/low level of cooking skills was associated with a low frequency of home cooking. Having low- or middle/low-level cooking skills was found to be significantly associated with high frequency of eating outside the home and with being underweight for men but not for women. The association between low or middle/low level of cooking skills and low frequency of vegetable/fruit intake was found for both men and women, but among men there was no dose–response relationship. The associations of low level of cooking skills with unhealthy dietary behaviors and underweight status were especially pronounced among men in charge of meals. Cooking skills were unassociated with obesity among both women and men.

In this study, a cooking skills scale for use in Japanese populations was designed with consideration of basic Japanese cooking methods and typical meals. Although we did not confirm the validity of this newly designed cooking skills scale by objective assessment, we were able to obtain plausible results, with the same trends observed with the original cooking skills scale for European populations [9]. Our cooking skills scale had appropriate internal consistency (Cronbach’s α = 0.96) and showed higher values for women and those with higher education levels, which is consistent with previous findings [9, 41]. This result also supports previous findings of a gender difference in confidence in cooking skills indicating that women are more confident in their cooking skills than are men [9, 22, 42]. The differences in cooking skills by gender and educational attainment among Japanese older adults may be explained by opportunities to learn cooking skills in school. In Japan, cooking education in schools was conducted exclusively for women until 1989; therefore, the men in this study (born before 1958) had less opportunity to learn cooking in school [43]. Another factor is that older age is associated with a stronger belief in the gender role ideology holding that men should work outside the home and women should do housework inside the home. In earlier years, there was even a cultural idea that men should not so much as enter the kitchen. This idea is reflected in the saying “*Danshi-chubo-ni-hairazu*” (“A man would be ashamed to be found in the kitchen”) [44]. However, men’s mean (SD) cooking skills score of 4.1 (1.42) was higher than the midpoint of 3.5, indicating that older Japanese men have above-average confidence in their cooking skills. In line with previous studies on adults showing that levels of cooking skills tend to be high in multiple-person households [8, 9], we found that women’s cooking skills were higher when they were married than when they were not. However, in contrast, men’s cooking skills were higher when they were not married (e.g., widowed or divorced). This result is intuitive because unmarried men do not have a spouse who is responsible for the cooking. We speculate that unmarried women have a moderate level of cooking skills because they were taught to cook in home economics classes and by their mothers, but their cooking skills may not have continued to improve because there was no need to cook for another person. Interventions earlier in the life course, such as at retirement, may be effective because men have a high risk of unhealthy eating behavior caused by their poor cooking skills if they are later widowed or divorced.

We included five basic cooking methods in the cooking skills scale, finding that men rated stewing and stir-frying as more difficult, compared with peeling and boiling. A previous study that examined eight cooking methods in the United Kingdom showed that men were more confident about boiling, compared with stewing or stir-frying [42]. This result is plausible because stewing and stir-frying require adjusting the level of heat and adding seasoning to prepare the dish properly. In interventions targeting men, it might be most beneficial to focus on simple cooking methods using stewing and stir-frying.

As expected, having a low or middle/low level of cooking skills was significantly associated with having a low frequency of home cooking for both men and women. People with high levels of cooking skills may enjoy cooking and feel self-confident regarding their cooking, leading to a high frequency of home cooking [9, 42]. However, a significant association between a low level of cooking skills and high frequency of eating outside the home was found only among men. This result may reflect the gender difference in the prevalence of eating outside the home. The percentage of respondents who ate out at least once a week was 15.5% for women and 23.6% for men (Table 2). A previous nationally representative survey in Japan reported that this gender difference exists across all age groups in the country, suggesting that men tend to prefer eating outside the home [45]. The same national survey also reported that the percentage of people who consume packaged lunches or cooked meals is the same for both men and women [45]. Therefore, women may consume these meals rather than eating outside the home, even if they have a low level of cooking skills. Another possible reason for the gender difference in eating outside the home is that there is a fundamental difference in cooking ability between men and women. In other words, the men who were categorized as having a low level of cooking skills cannot prepare any kind of meal, but the women who were categorized as having a middle/low level of cooking skills may be able to make basic meals. In our study, 79% of the women categorized as having a middle/low level of cooking skills were originally in the middle-level cooking skills category (Table 1). Therefore, women may not have to rely on eating outside the home even if they have a middle/low level of cooking skills.

Unlike home cooking and eating outside the home, no dose–response relationship between cooking skills and low vegetable/fruit intake was observed among men. In a previous study using the original cooking skills scale in Switzerland, favorable associations between cooking skills and various food groups including vegetables and fruits were evident for women, but these associations were weak or nonexistent for men [9]. This gender difference may be explained by nutritional knowledge [22, 46]: Men may not have sufficient nutritional knowledge regarding healthy food choice, even if they have a high level of cooking skills. Additionally, men may be more likely to choose foods because of their sensory appeal rather than for health reasons [47]. Future work should investigate food skills, including meal planning, food safety, and nutrition knowledge [23].

A low level of cooking skills was associated with underweight but not with obesity. Although a low level of cooking skills was associated with a high frequency of eating outside the home among men, which is generally associated with obesity, the majority of older Japanese people participating in this study did not rely on eating outside the home. The percentage of people who ate out more than three times a week was only 5% for the study participants, compared with 35% for older Americans aged 60 years or older [29]. In our sample, more than 90% of the participants reported that their daily meals were mainly cooked by themselves or a family member (Table 1). Therefore, for older Japanese people, a low cooking frequency because of a low level of cooking skills may mean that they skip meals or eat simple meals or meals with poor nutritional value instead of eating outside the home. This would be more likely to lead to underweight than to obesity. A study examining the amount of rice served at local and chain restaurants in Japan found that most restaurants set the rice potion at an appropriate quantity for middle-aged and older people (> 160 g and < 200 g) [48]. Therefore, those eating outside the home in Japan may be unlikely to consume a high number of calories. When we additionally included frequency of home cooking and vegetable/fruit intake as potential mediators in our model, the ARRR of underweight decreased and became statistically non-significant among men, although it remained significant among men in charge of meals (Supplementary Table 3, Model 2). Another possible explanation for the association between cooking skills and underweight is that cooking skills may be a surrogate indicator of physical capacity in daily living. To examine this hypothesis, we included limitations in instrumental activities of daily living status [49, 50] in our model as a confounding factor, confirming that the ARRR of underweight remained significant (Supplementary Table 3, Model 3). Contrary to expectations, a low level of cooking skills was associated with underweight but not with obesity among older adults. For Asian people, underweight is a consistent risk factor for death, and this risk is higher than that associated with obesity [51]. Underweight has also been reported to be associated with frailty [52], fracture, and bone loss [53], which are critical obstacles to maintaining quality of life among older people [54, 55]. Therefore, it may be important for health policy makers to identify people with poor cooking skills and organize programs to enhance cooking skills to prevent underweight.

The association between cooking skills and underweight was especially prominent among men in charge of meals, and this association remained significant in this group even after accounting for limitations in instrumental activities of daily living, frequency of home cooking, and vegetable/fruit intake (Supplementary Table 3, Model 4). This finding is plausible because many men have family members—often their wives—who prepare meals for them. Among the men in our study sample, 74% reported that a family member prepared their daily meals (Table 1). Considering that many men in charge of meals are widowed or divorced (Supplementary Table 2), these men may have difficulty preparing meals because they had few opportunities to prepare meals before losing their spouse. These men may thus be less motivated to cook and to eat, which can lead to a lower appetite [56]. Additionally, appetite decreases with age, and poor appetite has been shown to be related to, for example, lower intake of energy, protein, and vegetables/fruits; lower dietary diversity [57, 58]; and higher risks of malnutrition [59] and mortality [58]. Further studies are needed to examine the potential mechanisms of the risk of underweight caused by low levels of cooking skills for men. About half of older Japanese men who cook at home started cooking when they were over 50 years old [60]. Considering that cooking classes for men have increased over the past few decades in Japan, intervening at an older age may be feasible and acceptable.

This study had several limitations. First, we used self-reported weight and height to calculate BMI, which may have led to an under- or over-estimate of BMI [61]. A previous study demonstrated that, when calculated from self-reported weight and height, the BMI of older Japanese people was underestimated, compared with objective measures of weight and height; however, the same study showed that the BMI of underweight men and women was overestimated by 0.7 and 0.3, respectively, because these groups tended to over-report their weight [62]. Therefore, we may have underestimated the association of cooking skills with underweight. Second, we defined home cooking simply, excluding only the preparation of ready-to-eat food. Therefore, people who cooked low-quality meals or used some prepared foods in their cooking may have been included in the high frequency of home cooking category. This may have led to an underestimate of the association of cooking skills with low frequency of home cooking. Third, frequency of vegetable/fruit intake was assessed using a single, simple item. Future studies should use more detailed questions to assess which food groups are associated cooking skills. Fourth, we observed a ceiling effect for women’s cooking skills, as has been reported in a previous study using the original cooking scale [9]. Considering the reduction in the available time for cooking in recent years, it may be beneficial to investigate cooking skills not only in terms of methods (e.g., boiling and stewing), but also in terms of the ability to prepare a variety of meals in a short time. Moreover, a thorough psychometric assessment of the modified cooking scale should be performed if it continues to be used. More comprehensive, validated measures for assessing food and cooking skill confidence are now available [63]. Therefore, it is possible to use these measurement methods in the future. Fifth, we could only evaluate a limited number of eating behaviors. Future work should examine associations between cooking skills and other aspects of diet, such as dietary pattern, food components, and portion size, to understand the mechanisms of the relationship between cooking skills and weight status. Sixth, because the municipalities that participated in the JAGES survey were not randomly selected, the generalizability of our findings to other populations in Japan is limited. Finally, because this study was cross-sectional, we could not assess causality: Reverse causation is possible, and unmeasured factors such as personality may confound the examined associations. For example, underweight may be accompanied by frailty or low muscle strength, which may make it difficult to cook some foods or to cook for a long time while standing, resulting in low confidence in one’s cooking skills. However, more than half of the adult respondents in a previous study said that they had learned most of their cooking skills when they were teenagers and that they had learned these cooking skills mainly from their mothers [64]. Future randomized controlled trials comparing cooking, eating behaviors, and weight status among older adults with and without cooking skills interventions would clarify the causal association.

## Conclusions

Using a large-scale cross-sectional study, we confirmed that women had higher levels of cooking skills than did men and that the associations of cooking skills with dietary behaviors and weight status differed by gender. Moreover, the associations of cooking skills with unhealthy dietary behaviors and weight status were especially pronounced among men who were in charge of meals. Considering the possibility that the person in charge of meals may change in older age, research on support to improve cooking skills among older people is needed.

## Supplementary information

**Additional file 1: Table S1.** Adjusted relative risk ratios of underweight and overweight according to the cooking skills of older Japanese men (*n*=9,143) and women (*n*=10,595). **Table S2.** Characteristics of older Japanese men by cooking responsibility status (*n* = 9,203). **Table S3.** Adjusted relative risk ratios of underweight by cooking skill among older Japanese men.

## Data Availability

The datasets used and analyzed in the current study are from the JAGES study. All enquiries are to be addressed to the JAGES data management committee via e-mail: dataadmin.ml@jages.net. All JAGES datasets have ethical or legal restrictions for public deposition because of the inclusion of sensitive information from the human participants.

## Abbreviations

CI: confidence interval
BMI: body mass index
JAGES: Japan Gerontological Evaluation Study
