# Supplementary material for: Cooking skills related to potential benefits for dietary behaviors and weight status among older Japanese men and women: a cross-sectional study from the JAGES
Source: Int J Behav Nutr Phys Act. 2020 Jun 26;17:82. doi: 10.1186/s12966-020-00986-9 (PMC7318755; doi:10.1186/s12966-020-00986-9)
Supplement: Supplementary file 1 — Additional file 1: Table S1. Adjusted relative risk ratios of underweight and overweight according to the cooking skills of older Japanese men (n=9,143) and women (n=10,595). Table S2. Characteristics of older Japanese men by cooking responsibility status (n = 9,203). Table S3. Adjusted relative risk ratios of underweight by cooking skill among older Japanese men. [file 12966_2020_986_MOESM1_ESM.docx]

**Supplementary Table 1** Adjusted relative risk ratios of underweight and overweight according to the cooking skills of older Japanese men (n=9,143) and women (n=10,595).

|  |  |  | Underweight (< 18.5) |  | Overweight (≥ 23.0) |
| --- | --- | --- | --- | --- | --- |
|  |  |  | ARRR (95%CI) |  | ARRR (95%CI) |
| Men | | |  |  |  |
|  | Cooking skill | High | ref |  | ref |
|  |  | Middle | 1.24 (1.00 - 1.55) |  | 0.92 (0.84 - 1.01) |
|  |  | Low | 1.34 (0.99 - 1.81) |  | **0.86 (0.75 - 0.98)** |
| Women | | |  |  |  |
|  | Cooking skill | High | ref |  | ref |
|  |  | Middle-Low | 1.29 (0.98 - 1.68) |  | 0.97 (0.81 - 1.16) |

ARRR = adjusted relative risk ratio; BMI = body mass index; CI = confidence interval

The models were adjusted for age, education, annual normalized household income, marital status, and medical treatment (treatment for cancer, heart disease, stroke, diabetes mellitus, hypertension, and hyperlipidemia).

**Supplementary Table 2.** Characteristics of older Japanese men by cooking responsibility status (n = 9,203)

|  |  | Men in charge of preparing meals (n=2370) | |  | Men not in charge of preparing meals (n=6833) | | Person χ2 p-value |
| --- | --- | --- | --- | --- | --- | --- | --- |
|  |  | n | % |  | n | % |  |
| Cooking skill | |  |  |  |  |  |  |
|  | High | 1657 | 70.3 |  | 3094 | 45.6 | <0.001 |
|  | Middle | 572 | 24.3 |  | 2697 | 39.7 |  |
|  | Low | 129 | 5.5 |  | 994 | 14.6 |  |
| Age (years) | |  |  |  |  |  |  |
|  | 65–69 | 757 | 32.1 |  | 2098 | 30.9 | 0.03 |
|  | 70–74 | 599 | 25.4 |  | 1911 | 28.2 |  |
|  | 75–79 | 526 | 22.3 |  | 1533 | 22.6 |  |
|  | ≥80 | 476 | 20.2 |  | 1243 | 18.3 |  |
| Education (years) | |  |  |  |  |  |  |
|  | Low (≤9) | 760 | 32.2 |  | 1866 | 27.5 | <0.001 |
|  | Middle (10–12) | 858 | 36.4 |  | 2603 | 38.4 |  |
|  | High (≥13) | 712 | 30.2 |  | 2269 | 33.4 |  |
|  | Other/Missing | 28 | 1.2 |  | 47 | 0.7 |  |
| Annual income (million yen) | |  |  |  |  |  |  |
|  | Low (<2.00) | 939 | 39.8 |  | 2546 | 37.5 | <0.001 |
|  | Middle (2.00–3.99) | 746 | 31.6 |  | 2533 | 37.3 |  |
|  | High (≥4.00) | 188 | 8.0 |  | 736 | 10.8 |  |
|  | Missing | 485 | 20.6 |  | 970 | 14.3 |  |
| Marital status | |  |  |  |  |  |  |
|  | Married | 1273 | 54.0 |  | 6515 | 96.0 | <0.001 |
|  | Widowed | 469 | 19.9 |  | 162 | 2.4 |  |
|  | Divorced | 303 | 12.8 |  | 18 | 0.3 |  |
|  | Not married | 239 | 10.1 |  | 18 | 0.3 |  |
|  | Other/Missing | 74 | 3.1 |  | 72 | 1.1 |  |
| Under medical treatment | |  |  |  |  |  |  |
|  | Cancer (Yes) | 108 | 4.6 |  | 342 | 5 | 0.002 |
|  | Heart disease (Yes) | 319 | 13.5 |  | 900 | 13.3 | 0.002 |
|  | Stroke (Yes) | 82 | 3.5 |  | 303 | 4.5 | <0.001 |
|  | Diabetes mellitus (Yes) | 392 | 16.6 |  | 1092 | 16.1 | 0.001 |
|  | Hypertension (Yes) | 999 | 42.4 |  | 2914 | 42.9 | 0.002 |
|  | Hyperlipidemia (Yes) | 206 | 8.7 |  | 727 | 10.7 | <0.001 |
| Frequency of home cooking (n/week) | | |  |  |  |  |  |
|  | ≥5 /week | 1068 | 45.3 |  | 242 | 3.6 | <0.001 |
|  | 3-4 /week | 395 | 16.8 |  | 362 | 5.3 |  |
|  | 1-2 /week | 272 | 11.5 |  | 730 | 10.8 |  |
|  | < 1 /week | 164 | 7 |  | 993 | 14.6 |  |
|  | Never | 459 | 19.5 |  | 4458 | 65.7 |  |
| Frequency of eating out (n/week) | | | |  |  |  |  |
|  | ≥5 /week | 106 | 4.5 |  | 136 | 2 |  |
|  | 3-4 /week | 170 | 7.2 |  | 292 | 4.3 |  |
|  | 1-2 /week | 409 | 17.3 |  | 1041 | 15.3 |  |
|  | < 1 /week | 773 | 32.8 |  | 3000 | 44.2 |  |
|  | Never | 900 | 38.2 |  | 2316 | 34.1 |  |
| Frequency of vegetable/fruit intake (n/day) | | | | | | |  |
|  | ≥1 /day | 1451 | 61.5 |  | 5174 | 76.3 | <0.001 |
|  | <1 /day (low frequency) | 907 | 38.5 |  | 1611 | 23.7 |  |
| Body weight status (BMI, kg/m^2^) | | | | | | |  |
|  | Underweight (< 18.5) | 115 | 4.9 |  | 313 | 4.6 | 0.83 |
|  | Normal (18.5–27.4) | 2067 | 87.7 |  | 5977 | 88.1 |  |
|  | Obesity (≥ 27.5) | 176 | 7.5 |  | 495 | 7.3 |  |

BMI = body mass index

**Supplementary Table 3.** Adjusted relative risk ratios of underweight by cooking skill among older Japanese men

|  |  |  | Model 1 |  | Model 2 |  | Model 3 |  | Model 4 |
| --- | --- | --- | --- | --- | --- | --- | --- | --- | --- |
|  |  |  | ARRR (95%CI) |  | ARRR (95%CI) |  | ARRR (95%CI) |  | ARRR (95%CI) |
| Men (n=9,143) | | |  |  |  |  |  |  |  |
|  | Cooking skill | High | ref |  | ref |  |  |  | ref |
|  |  | Middle | **1.29 (1.04 - 1.60)** |  | 1.19 (0.94 - 1.50) |  | **1.25 (1.01 - 1.56)** |  | 1.17 (0.92 - 1.47) |
|  |  | Low | **1.43 (1.06 - 1.92)** |  | 1.30 (0.94 - 1.80) |  | 1.31 (0.95 - 1.80) |  | 1.22 (0.86 - 1.71) |
| Men in charge of cooking (n=2,358) | | |  |  |  |  |  |  |  |
|  | Cooking skill | High | ref |  | ref |  | ref |  | ref |
|  |  | Middle | **1.59 (1.04 - 2.45)** |  | 1.49 (0.93 - 2.37) |  | 1.62 (1.05 - 2.50) |  | 1.50 (0.94 - 2.39) |
|  |  | Low | **2.79 (1.45 - 5.36)** |  | **2.64 (1.27 - 5.48)** |  | **2.95 (1.50 - 5.82)** |  | **2.73 (1.30 - 5.73)** |

ARRR = adjusted relative risk ratio; CI = confidence interval

Model 1: Adjusted for age, education, annual normalized household income, marital status, and medical treatment (treatment for cancer, heart disease, stroke, diabetes mellitus, hypertension, and hyperlipidemia)

Model 2: Model 1 + adjusted for frequency of home cooking and vegetable/fruit intake

Model 3: Model 1 + adjusted for limitations in instrumental activities of daily living (IADL)

Model 4: Model 1 + adjusted for frequency of home cooking and vegetable/fruit intake and limitations in IADL

Assessment of limitations in IADL used the Tokyo Metropolitan Institute of Gerontology Index of Competence, which comprises the following five items: “Can you use public transportation (bus or train) by yourself?”; “Are you able to shop for daily necessities?”; “Are you able to prepare meals by yourself?”; “Are you able to pay bills?”; and “Can you handle your own banking?” These items have a *yes* or *no* response format (Koyano, Shibata, Nakazato, Haga, & Suyama, 1991).
